# Effectiveness of digital co-creation platform in remote pulmonary rehabilitation for older adults with chronic obstructive pulmonary disease: a randomized controlled trial

**DOI:** 10.3389/fpubh.2025.1708607

**Published:** 2025-11-10

**Authors:** Xueying Huang, Xianjing Song, Manyao Sun, Yi Hou, Jiang Nan, Jing Gao, Songxin Fu, Yuyu Jiang

**Affiliations:** Department of Nursing, Wuxi School of Medicine, Jiangnan University, Wuxi, Jiangsu, China

**Keywords:** chronic obstructive pulmonary disease, randomized controlled trial, co-creation, the CoI framework, telemedicine

## Abstract

**Aims:**

Despite proven benefits, older adults with chronic obstructive pulmonary disease (COPD) face significant challenging in adhering to telehealth-enabled pulmonary rehabilitation (Tele-PR). Co-creation represents an innovative approach to behavioral intervention. The aim of this study is to investigate the effect of improving activation and adherence among older adults with COPD who accepted an intervention incorporating co-creation sessions.

**Methods:**

A total of 102 older adults diagnosed with COPD were randomly assigned to the control group (PR group) or digitalization co-creation group (CC-CoI group). The intervention lasted for 12 weeks, and the follow-up was 12 weeks. The primary outcomes were patient activation and Tele-PR adherence. The secondary outcomes included dyspnea symptoms, exercise self-efficacy, self-management ability, social support, cognitive level, and satisfaction with medical service. Data were collected at baseline, week 12, and week 24.

**Results:**

A total of 102 participants completed the study. At weeks 12 and 24, there were statistically significant differences in patient activation (*p* < 0.001, *p* < 0.001) between the two groups. Tele-PR adherence remained effectively maintained in the CC-CoI group (*p* = 0.607, *p* = 0.332), while the PR group showed a significant decline (*p* = 0.006, *p* < 0.001). The CC-CoI group also exhibited significant improvements in the modified Medical Research Council (mMRC) scale score, Exercise Self-Regulatory Efficacy Scale (Ex-SRES) score, COPD Self-Management Scale (CSMS) score, Perceived Social Support Scale (PSSS) score, and COPD knowledge.

**Conclusion:**

These results indicate that digital co-creation enhances the active participation of older adults in Tele-PR and fosters the development of virtual communities along with the onset of altruistic behaviors in individuals. This approach encourages patients to apply their knowledge for self-management of health behaviors, which in turn, enhances their adherence to rehabilitation exercise and leads to better health outcomes.

**Clinical trial registration:**

Chinese Clinical Trial Registry (ChiCTR): ChiCTR1900028563/December 27, 2019. http://apps.who.int/trialsearch/default.aspx.

## 1 Introduction

Chronic obstructive pulmonary disease (COPD) is a prevalent and costly respiratory disease (1), projected to become the third leading cause of death and the fifth most significant economic burden globally by 2030 (2, 3). With the aging global population, the proportions of patients with COPD over the age of 65 is increasing, affecting an estimated 20–25% of this demographic (4, 5).

Pulmonary rehabilitation (PR), with exercise training combined with disease-specific education as the core components, is widely recognized as a fundamental part of COPD treatment and management (6). Telehealth-enabled pulmonary rehabilitation (Tele-PR) refers to the delivery of rehabilitation services to home-based patients remotely using information and communication technologies, such as smart wristbands, actigraphy, and tablet personal computer (7, 8). With advancements in information technology, Tele-PR is gaining popularity due to its convenience, cost-effectiveness, and broad accessibility (9). With the potential to improve acceptance and access to PR, Tele-PR could alleviate symptoms, enhance activity tolerance, improve quality of life, and reduce the strain on healthcare systems (10). Despite its benefits, adherence to Tele-PR among older adults with COPD remains challenging (11, 12). A meta-analysis by Alghamdi et al. (13) revealed that older adults with COPD showed a high level of acceptance of the Tele-PR clinical trial, with participation rates of 80 and 51% for unweighted and weighted participants, respectively. However, participation rates declined in large-scale scenarios, and the withdrawal rate in the Tele-PR group (63%) was significantly higher than in the control group (37%) (13). In a 3-month Tele-PR program for older adults with COPD conducted by Jiang et al. (14), nearly half of the patients exhibited low adherence, which dropped from 58.3 to 33.3%. Studies have shown that improvements in patient health are associated with adherence to Tele-PR; cessation of exercise training can lead to a loss of recovery benefits within a year (15).

Patient activation is critical for participation and adherence to Tele-PR, yet older adults with COPD often lack this engagement (16, 17). The reasons for insufficient patient activation may include limited patient understanding and awareness of their disease condition and the practical effects of exercise rehabilitation (18), as well as their inability to engage in the implementation of Tele-PR programs, which rely on a traditional expert-driven model (19). Currently, researchers have primarily employed Social cognitive theory and behavioral interventions as the main strategies to boost patient activation (20–22).

Co-creation is a novel strategy for behavioral intervention. Co-creation in research is broadly defined as the collaborative generation of knowledge, incorporating diverse stakeholders perspectives (23), which has the potential to address the issue of lack of activation (24, 25). With advancements in information and digital technologies, co-creation enables the integration of resources from all stakeholders via interactive platforms, facilitating value creation and the generation of innovative outcomes (26, 27). This process can be viewed as a form of digital labor (28). Through co-creation, patients transition from passive recipients to active participants in medical decision-making and nursing practices, thereby enhancing their disease management capabilities and adherence to exercise rehabilitation (17, 22, 24). Furthermore, the co-creation strategy can optimize patient education, improve patients’ understanding of disease management, and facilitate doctor-patient communication, ultimately enhancing treatment outcomes (29, 30). Consequently, this study integrated the co-creation phase into the implementation of the Tele-PR program to boost patient activation and adherence.

Additionally, several studies have demonstrated that the successful implementation of co-creation requires the guidance of a theoretical framework (31, 32). The Community of Inquiry (CoI) framework is a collaborative-constructivist framework that comprises three critical elements: teaching presence, social presence, and cognitive presence (33–35). It provides theoretical guidance for various aspects of co-creation, including knowledge preparation, social interaction, and knowledge application. This framework emphasizes the transfer of knowledge and skills throughout the practical process. Through interaction, collaboration, and continuous reflection, participants progressively deepen their understanding while fostering the sharing and application of knowledge. This process ensures that all stakeholders engage in genuine co-creation (36, 37), thereby achieving the goal of co-creation, aligning with value co-creation, value sharing, and value co-winnings (38). To date, no transformation studies applying the CoI framework to COPD management have been published.

This study aimed to incorporate co-creation into the Tele-PR intervention process. By utilizing the CoI framework, we developed and evaluated a “digital co-creation” (CC-CoI) platform, which was applied over a 12-week intervention period, followed by an additional 12-week follow-up to verify its effectiveness in improving the activation and adherence of older adults with COPD.

## 2 Methods

### 2.1 Study design

This study was a 24-week randomized controlled trial with a 12-week standardized Tele-PR intervention followed by a 12-week rehabilitation observation period. The remote intervention utilizes WeChat and the Pulmonary Internet Explorer Rehabilitation (PeR), a free WeChat public account previously developed by our research group (39). After completing the baseline assessment, patients were randomly assigned in a 1:1 ratio to either the intervention (CC-CoI group) or control group (PR group). The random-assignment sequence was generated using computer software and managed by an independent research assistant who was not involved in participant recruitment, evaluation, or intervention. Data collection at baseline, 12 weeks, and 24 weeks was conducted by research assistants who were blinded to group assignments. For this study, the same assessor was responsible for collecting both the baseline and follow-up data. The study was approved by the ethical review of Jiangnan University (JNU20220310IRB17) and registered with the Chinese Clinical Trial registry [ChiCTR1900028563]. All patients signed the paper-based informed consent form, and the checklist can be found in the Supplementary Table 1.

### 2.2 Participants and recruitment procedure

From September 1, 2023, to August 1, 2024, patients were recruited from the five hospitals in Wuxi using leaflets, posters, and face-to-face communication. The participants in this study received no rehabilitation treatment beyond the planned intervention. Eligible participants were 65 years and older, diagnosed with COPD based on established guidelines (FEV1/FVC ratio <0.7, FEV1 < 80% predicted (40)), and in a stable stage of the disease. Participants needed to use WeChat for communication. Exclusion criteria included mental disorders, cognitive impairment, limb dysfunction, unstable heart conditions, recent myocardial or cerebral infarction, inability to perform muscle strength tests, uncontrolled hypertension, or a history of syncope after exercise. Informed consent was obtained from all participants, who retained the right to withdraw at any time. Baseline data, including demographic, clinical information, and primary and secondary outcome measures, were collected by community physicians, nurses, and research assistants.

### 2.3 Sample size

The primary outcome of this study is patient activation, which is measured using the Patient Activation Measure (PAM-13). With the test level of 0.05 on both sides and 80% test efficacy, the sample size estimation method was used for each group’s measurement data. Drawing on existing research, the effect size for the PAM-13 was set at 0.79 (41–44). When the sample number of each group was equal, at least 36 cases were needed in each group. According to the estimation of 13% dropout rate in the study (39), at least 41 cases were needed in each group, and at least 82 cases were included in the sample size. This study involved the simultaneous recruitment of participants across five hospitals. Given the ongoing interest expressed by patients during this period, and in order to respect their willingness and protect their rights, the study allowed these patients to continue participating in the intervention. The final sample size was 102.

### 2.4 Development of the CC-CoI platform

**Figure 1:**
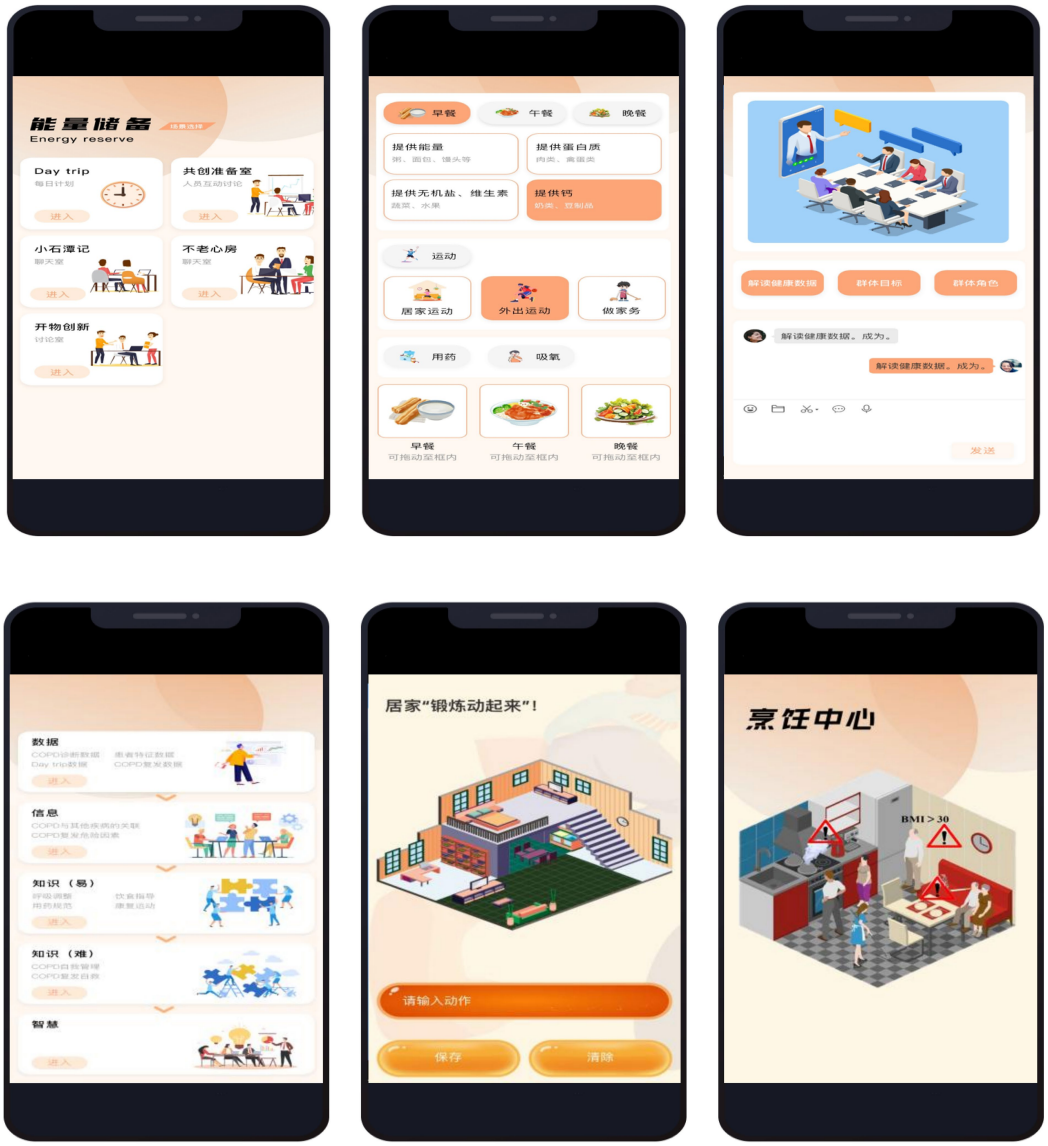
Part of the interface of the CC-CoI platform.

This study develops CC-CoI platform based on the existing PeR. PeR has an “energy zone” component, which can be expanded to realize different interventions with different characteristics. In this study, the CC-CoI platform was added to the “energy zone” component. Within the present study the first four phases (0–3) of the mHealth Agile Development & Evaluation Lifecycle were completed (45). Phase Zero (Project Identification Phase): A focus group consisting of respiratory therapy specialists, clinicians, rehabilitation practitioners, nurses, software engineers, user interface designers, and older adults with COPD was formed. They conducted interviews to understand the obstacles of participants’ low adherence with Tele-PR programs and key expectations for the platform. Phase One (Development and Alpha Testing): This study developed the functional modules of CC-CoI platform according to the features of the CoI framework components (33), including the ‘Small Tarn West of the Knoll’, ‘Ageless Atrium’, and ‘Exploitation of Innovation’. Through focused interviews with older adults with COPD and healthcare professionals (HCPs), four themes for co-creating comics in the ‘Ageless Atrium’ module were identified. Subsequently, an image gallery (including images with correct and incorrect meanings) was constructed based on the thematic comic scene-keyword mapping chart to activate the text-to-image generation feature. The ‘Exploitation of Innovation’ module’s four virtual scenarios were tailored according to the Instrumental Activities of Daily Living (IADLs) (46) and the St. George’s Respiratory Questionnaire (SGRQ) (47). As a result of the Alpha testing, we added the ‘Co-creation Discussion Room’ and ‘Day trip’ function modules. Phase Two (Beta testing): The results showed that the task completion rate of CC-CoI platform reached 100%, which indicated the complete feasibility of functional design. The CES average of 3.30 and the NPS average of 4.60 reflected high operational efficiency and user satisfaction. The specific content of the Alpha and Beta testing and program design is provided in Supplementary Table 2. Patients can see the part of the interface of the CC-CoI platform (Figure 1). In phase three, the platform’s intervention effectiveness was validated through a randomized controlled trial. In phase four, this study has not yet conducted post-market surveillance of the product.

### 2.5 Intervention

#### 2.5.1 PR group

A 12-week Tele-PR program, pre-validated by the research team, was adopted (39). This program was independently designed and subsequently verified for validity. The PR group was unable to access any of the other functions within the “energy zone” component. The core part of the intervention program included exercise training, respiratory training, medication knowledge, and diet guidance. Nurses assessed the patient’s condition and tailored a personalized exercise prescription. They instructed patients on how to register and log in to PeR, access evidence-based PR videos and text materials, complete and upload self-assessment reports, and communicate with peers and nurses through PeR to share experiences. Additionally, nurses maintained weekly contact with patients via phone or WeChat, providing professional guidance and advice based on the self-assessment reports uploaded by patients. If patients experienced acute deterioration in their condition, they could contact the nurses promptly through the online platform, and nurses would offer guidance based on the situation. The patient’s exercise situation was summarized by the nurses’ feedback every 4 weeks.

#### 2.5.2 CC-CoI group

The CC-CoI group received not only the same Tele-PR intervention components as the PR group, but also a nurse-led CC-CoI platform intervention consisting of five modules:

1. ‘Day trip’: After logging into PeR, the nurse assisted patients to create their own virtual cartoon image and mark their daily schedule, including home-cooked meals, medication and exercise, etc.
2. ‘Small Tarn West of the Knoll’: The platform adopted the visual learning map to implement a three-week educational program on PR knowledge. In week 1, the nurse explained the meaning of medical data (physiological indicators and diagnostic criteria) and information about the disease (risk factors for recurrence and complications). In weeks 2 and 3, the nurse guided the patient to improve self-management skills (respiratory training, nutrition care, medication knowledge and management, rehabilitation exercise, identification of acute episodes and effective communication with HCPs, etc.) according to ‘Day trip’ module.
3. ‘Co-creation Discussion Room’: In week 4, the nurse helped the patient to review the previous content, introduced the function of the ‘Ageless Atrium’ module, taught the patient how to use it, and provided a thematic comic scene-keyword mapping chart.
4. ‘Ageless Atrium’: Completed in week 4. The nurse facilitated a group of 4–5 older adults with COPD to sequentially co-create four thematic comics (“Living with COPD,” “Exercise to Move,” “Safety Lessons from the Medicine Cabinet” and “COPD and Caring for Your Heart”), thereby completing digital labor and achieving digital socialization. The process was as follows: Through questioning, nurses guided patients to enter the keywords of PR knowledge according to their own rehabilitation experience and understanding, respectively. The platform automatically generated images, which could be integrated into a single background image called a draft image. The nurse organized the patients to express their understanding of pulmonary rehabilitation knowledge using the draft images. During this process, both the nurse and the doctor could correct the patients’ misconceptions and judgments of PR knowledge and guide them to jointly create a comic with an accurate theme. Doctors recorded shareable 30-s audio health education clips by long-pressing the microphone button. When patients’ lung rehabilitation knowledge input error rate exceeded 40%, the system automatically restarted the digital practice for that topic. The software engineers provided a clickable area for each generative image and linked to the corresponding PeR video material, making it easy for patients to click through for review and rehabilitation exercise. The demonstration video is attached in Supplementary Video 1.
5. ‘Exploitation of Innovation’: Completed in week 4. There were four virtual scenarios (Eco-serenity Oasis, Vogue Plaza Delight, Culinary Innovation Hub and Living Sphere) to train patients in identifying the risk factors for COPD recurrence. Each scenario contained 3 correct cartoon images of risk factors and 2 incorrect cartoon images. If the patient moved the cartoon images of the risk factor into the trash bin within 3 min, the task was considered complete. If the patient was unable to complete the action, the platform automatically removed the cartoon images of the risk factor. Risk factors and task settings are attached in Supplementary Table 2.

In weeks 8 and 12, the nurse guided the patients to repeat the training of the ‘Small Tarn West of the Knoll’, ‘Ageless Atrium’, and ‘Exploitation of Innovation’ modules. Depending on the patients’ needs, the nurse reviewed the patients’ exercise situation every 4 weeks, either in person or via WeChat video.

### 2.6 Outcome measures

#### 2.6.1 Primary outcomes

Because CC-CoI platform aims to prepare and to activate individuals to take a role in adhering to Tele-PR program, we chose patient activation and adherence to Tele-PR as the primary outcomes. Patient activation was assessed using the PAM-13 (range 0–100). The PAM-13 measures individuals’ knowledge, skills, and confidence to manage their health across 4 levels of activation (levels 1–4), with higher scores indicating a higher activation level and a minimally important difference of 4 points (43, 48). Tele-PR adherence refers to the extent to which patients follow their prescribed exercise rehabilitation plan and how closely their adherence aligns with the plan developed by healthcare professionals. Specifically, Tele-PR adherence was collected through patients’ self-reports, and it was classified according to whether the proportion between the actual completed rehabilitation exercise and the plan reached 75%. Adherence was classified into high adherence and low adherence (49).

#### 2.6.2 Secondary outcomes

Symptoms and disease effects were assessed using the CAT, which consists of 8 items. A higher total score indicates worse health status (50, 51). Furthermore, the Minimal Clinically Important Difference (MCID) is defined as a decrease of ≥2 points in the CAT score and serves as a key indicator for assessing treatment response in older adults with COPD (52). Dyspnea was evaluated using the mMRC scale, ranging from 0 to 4, with higher scores indicating more severe dyspnea (53). Exercise self-efficacy was measured by the Ex-SRES, where higher score reflects greater confidence in maintaining exercise (54). Self-management ability was assessed using the CSMS, which includes 51 items, with higher scores indicating better self-management capacity (55, 56). Social support was measured using the PSSS, widely used in China, where a higher score indicates a higher perceived level of social support (57). Cognitive level was assessed using the COPD-Q, with higher accuracy rates representing better disease knowledge (58). Satisfaction with healthcare service was measured using a self-administered, single-item, 11-point Likert scale with the question: ‘How would you rate our services?’. The higher the score, the higher the patient’s satisfaction with the medical service.

### 2.7 Data collection and analysis

Patient demographics and baseline outcome measures were collected before the intervention (T0), with adherence to Tele-PR was measured in the first week (T1w). All outcome measures were collected at 12 and 24 weeks (T1, T2) of the intervention period.

An intention-to-treat analysis was employed, using the last observation carried forward method to handle missing data. Data analysis was conducted using SPSS Statistics version 27.0, with statistical significance set at *p* < 0.05. Baseline characteristics between the groups were compared using t-test or Mann–Whitney U tests, and categorical variables were analyzed using *χ*^2^ test. Changes in PAM-13, CAT, Ex-SRES, CSMS, PSSS, and COPD-Q scores over different intervention time points (T0, T1 and T2) were analyzed using repeated measures ANOVA, with significant interaction effects further examined through simple effects analysis. The mMRC score was analyzed using the Friedman test, with significant changes analyzed by Wilcoxon signed-rank tests. Between-group differences at various intervention time points (T1–T0, T2–T0) were evaluated using the Mann–Whitney *U* test, and exercise adherence in the Tele-PR group was compared using *χ*^2^ and McNemar tests. In addition, the difference in satisfaction with medical services between the two groups at the end of the 12-week intervention (T1) was compared using the Mann–Whitney *U* test.

## 3 Results

### 3.1 Basic demographic characteristics and measurements

**Figure 2:**
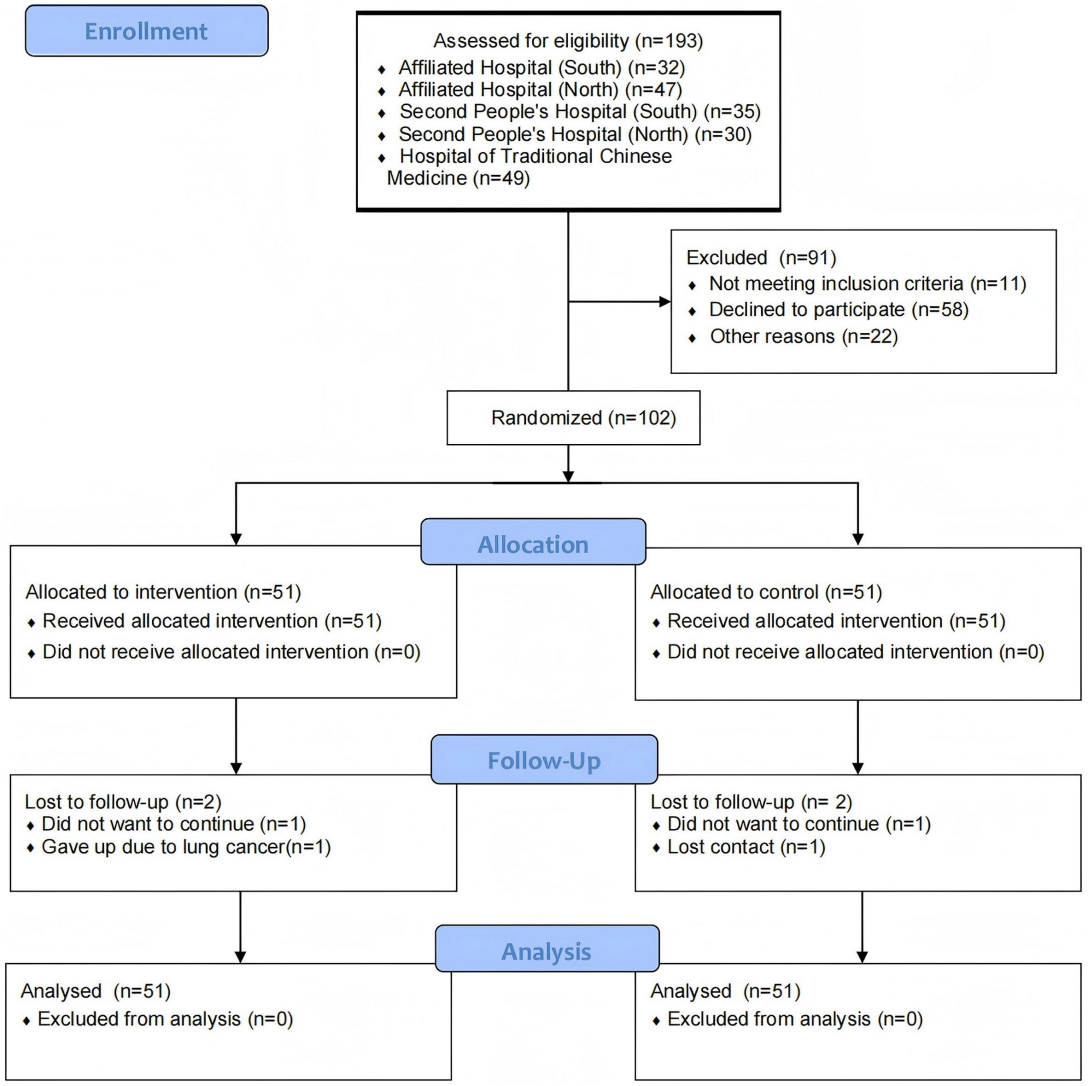
Consolidated Standards of Reporting Trials flowchart.

Recruitment for this study commenced on September 1, 2023, with follow-ups completed by August 1, 2024. Figure 2 shows the CONSORT flowchart. The study recruited 102 older adults with COPD, randomly assigned to the CC-CoI group (*n* = 51) or the PR group (*n* = 51). At baseline, both groups were comparable in terms of demographic characteristics and outcome measures (*p* > 0.05), as presented in Table 1.

**Table 1:** Comparison of baseline information between the two groups.

Measures	IG (*n* = 51)	CG (*n* = 51)	$\chi^{2}$ /*Z*/t	*P*
	Mean (SD)/Median (range)/*n*%			
Sex			0.000	>0.999^b^
Male	44(86)	44(86)		
Female	7(14)	7(14)		
Age, years	73.31 ± 6.16	74.92 ± 4.96	−1.453	0.149^a^
BMI	23.76 ± 2.77	23.01 ± 2.10	1.557	0.123^a^
Disease duration, years			0.423	0.809^b^
<5	11(21)	13(25)		
5–10	19(37)	20(39)		
≥10	21(42)	18(36)		
GOLD classification			1.524	0.467^b^
GOLD II grade	26(51)	22(43)		
GOLD III grade	18(35)	24(47)		
GOLD IV grade	7(14)	5(10)		
Education			0.744	0.946^b^
Primary school	10(19)	11(22)		
Middle school	20(39)	23(45)		
High school	8(16)	6(12)		
Technical school	9(18)	8(16)		
College or postgraduate	4(8)	3(5)		
Monthly income, RMB, ¥			0.197	0.657^b^
<5,000	36(71)	38(75)		
≥5,000	15(29)	13(25)		
Marital status			0.249	0.618^b^
Married	42(82)	40(78)		
Never married / divorced / widowed	9(18)	11(22)		
Social status (living alone)			0.331	0.565^b^
Yes	8(16)	6(12)		
No	43(84)	45(88)		
Smoking status			0.343	0.842^b^
Never smoked	9(18)	9(18)		
Former smoker	34(66)	36(70)		
Current smoker	8(16)	6(12)		
Cumulative smoking, years			3.374	0.185^b^
<20	10(20)	9(18)		
20–40	9(18)	17(33)		
>40	32(62)	25(49)		
Hospitalizations in the past year			1.520	0.678^b^
None	8(16)	7(14)		
One time	27(53)	25(49)		
Two times	12(24)	11(22)		
Three or more times	4(7)	8(15)		
PAM-13 score	54.35 ± 6.54	54.64 ± 6.63	−0.219	0.827^a^
CAT score	21.76 ± 3.64	21.18 ± 4.16	0.760	0.449^a^
mMRC score	2(2,3)	2(2,3)	−0.952	0.341^c^
Ex-SRES score	70.75 ± 4.45	71.14 ± 4.79	−0.429	0.669^a^
CSMS score	134.43 ± 9.95	134.69 ± 7.62	−0.145	0.885^a^
PSSS score	57.27 ± 4.83	56.71 ± 5.04	0.582	0.562^a^
COPD-Q score	6.45 ± 1.62	6.14 ± 1.84	0.914	0.363^a^

PAM-13, Patient Activation Measure; CAT, COPD Assessment Test; mMRC, modified Medical Research Council scale; Ex-SRES, Exercise Self-Regulatory Efficacy Scale; CSMS, COPD Self-Management Scale; PSSS, Perceived Social Support Scale; COPD-Q, COPD Knowledge Questionnaire.

^a^Independent Samples *t*-test; ^b^Chi-squared test; ^c^Mann–Whitney *U* test.

### 3.2 Primary outcome

**Figure 3:**
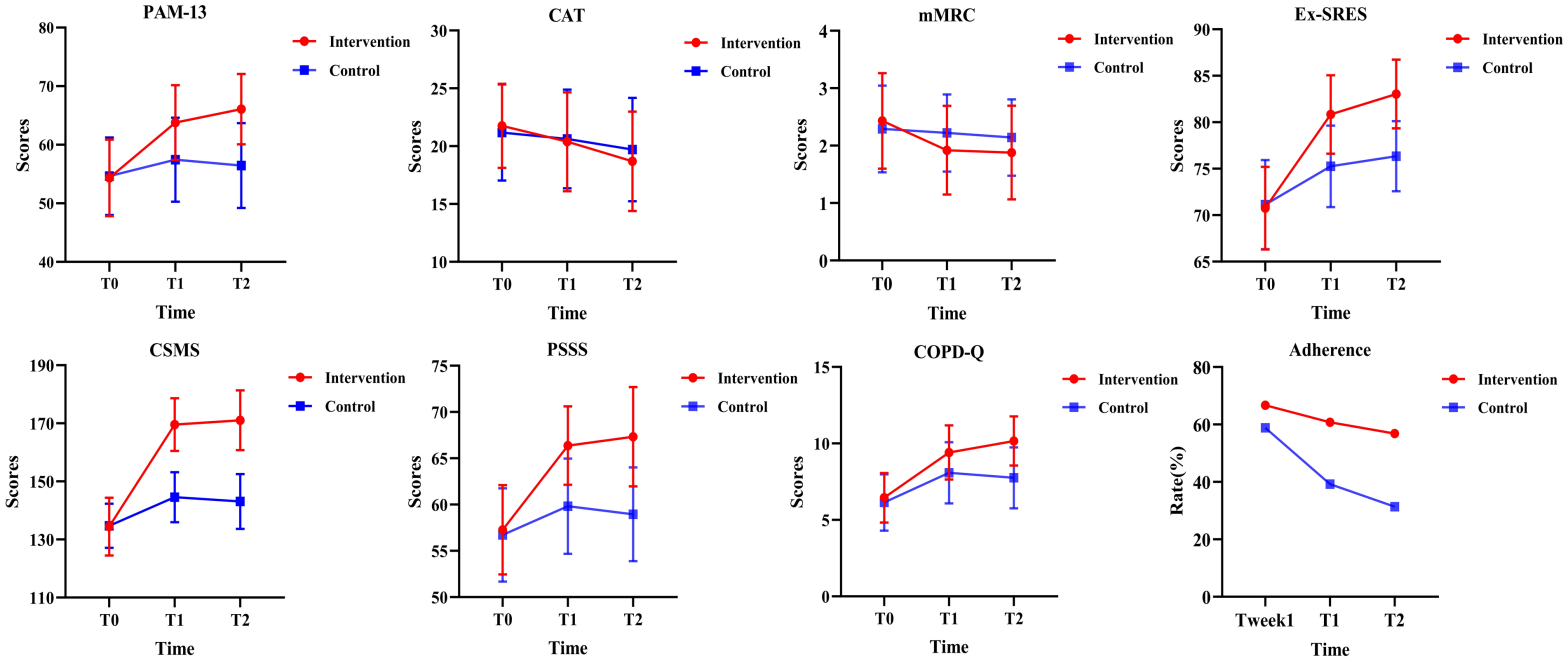
Evaluation indicators and their change over time.

The CC-CoI group exhibited a significant increase in PAM-13 scores, rising by 11.74 points by 24 weeks post-intervention (*p* < 0.001), compared to a non-significant increase of 1.82 points in the PR group (*p* = 0.166). This difference was significant at both the week 12 (*F* = 23.01, *p* < 0.001, *η*^2^ = 0.32) and week 24 (*F* = 50.11, *p* < 0.001, *η*^2^ = 0.50) (Table 2), with a significant group-by-time interaction effect observed (*F* = 42.77, *p* < 0.001, *η*^2^ = 0.46) (Table 3). The trend in PAM-13 scores over time is shown in Figure 3.

**Table 2:** Means, standard deviations, and two-way ANOVA statistics for study variables.

Measures	Mean (SD)				ANOVA			
	Intervention		Control		Effect	*F* ratio	*P*	*η* ^2^
PAM-13								
T0	54.35	6.54	54.64	6.63	G	20.994	<0.001	0.296
T1	63.79	6.40	57.46	7.16	T	101.405	<0.001	0.670
T2	66.09	5.99	56.46	7.23	G × T	42.769	<0.001	0.461
CAT								
T0	21.76	3.64	21.18	4.16	G	0.146	0.704	0.003
T1	20.39	4.28	20.63	4.27	T	18.650	<0.001	0.272
T2	18.69	4.31	19.71	4.47	G × T	2.302	0.105	0.044
Ex-SRES								
T0	70.75	4.45	71.14	4.79	G	29.532	<0.001	0.371
T1	80.84	4.22	75.25	4.39	T	369.030	<0.001	0.881
T2	83.04	3.69	76.35	3.77	G × T	66.937	<0.001	0.572
CSMS								
T0	134.43	9.95	134.69	7.62	G	105.998	<0.001	0.679
T1	169.57	9.05	144.59	8.60	T	544.783	<0.001	0.916
T2	171.06	10.28	143.08	9.43	G × T	169.058	<0.001	0.772
PSSS								
T0	57.27	4.83	56.71	5.04	G	1691.030	<0.001	0.971
T1	66.37	4.24	59.82	5.15	T	1089.704	<0.001	0.956
T2	67.33	5.37	58.94	5.06	G × T	397.960	<0.001	0.888
COPD-Q								
T0	6.45	1.62	6.14	1.84	G	22.031	<0.001	0.306
T1	9.41	1.77	8.08	2.00	T	86.050	<0.001	0.632
T2	10.16	1.61	7.75	2.00	G × T	11.294	<0.001	0.184

T0, baseline survey; T1, 12-week; T2, 24-week; PAM-13, Patient Activation Measure; CAT, COPD Assessment Test; Ex-SRES, Exercise Self-Regulatory Efficacy Scale; CSMS, COPD Self-Management Scale; PSSS, Perceived Social Support Scale; COPD-Q, COPD Knowledge Questionnaire.

**Table 3:** Within-group differences and between-group differences of outcomes.

Measures	Difference at 3 months						Difference at 6 months					
	Within group		Between group				Within group		Between group			
	Mean (95%CI)	*P*	Mean (95%CI)	*P*	*F*	*η* ^2^	Mean (95%CI)	*P*	Mean (95%CI)	*P*	*F*	*η* ^2^
PAM-13												
I	9.44 (7.82 to 11.05)	<0.001	NA	NA	NA	NA	11.74(9.95 to 13.54)	<0.001	NA	NA	NA	NA
C	2.82(0.72 to 4.92)	0.005	NA	NA	NA	NA	1.82(−0.48 to 4.11)	0.166	NA	NA	NA	NA
I vs. C	NA	NA	6.33(3.68 to 8.98)	<0.001	23.012	0.315	NA	NA	9.64(6.90 to 12.38)	<0.001	50.108	0.501
CAT												
I	−1.37(−2.18 to −0.56)	<0.001	NA	NA	NA	NA	−3.08(−3.94 to −2.21)	<0.001	NA	NA	NA	NA
C	−0.55(−1.78 to 0.68)	0.822	NA	NA	NA	NA	−1.47(−3.59 to 0.65)	0.276	NA	NA	NA	NA
I vs. C	NA	NA	−0.24(−1.80 to 1.33)	0.764	0.091	0.002	NA	NA	−1.02(−2.08 to 0.04)	0.058	3.750	0.070
Ex-SRES												
I	10.10(8.57 to 11.62)	<0.001	NA	NA	NA	NA	12.29(10.58 to 14.01)	<0.001	NA	NA	NA	NA
C	4.12(3.33 to 4.91)	<0.001	NA	NA	NA	NA	5.22(4.28 to 6.16)	<0.001	NA	NA	NA	NA
I vs. C	NA	NA	5.59(3.82 to 7.35)	<0.001	40.497	0.447	NA	NA	6.69(5.22 to 8.15)	<0.001	84.011	0.627
CSMS												
I	35.14(31.20 to 39.08)	<0.001	NA	NA	NA	NA	36.63(32.31 to 40.94)	<0.001	NA	NA	NA	NA
C	9.90(7.47 to 12.33)	<0.001	NA	NA	NA	NA	8.39(5.78 to 11.00)	<0.001	NA	NA	NA	NA
I vs. C	NA	NA	24.98(21.19 to 28.77)	<0.001	175.345	0.778	NA	NA	27.98(23.77 to 32.19)	<0.001	178.203	0.781
PSSS												
I	9.10(8.61 to 9.59)	<0.001	NA	NA	NA	NA	10.06(9.39 to 10.73)	<0.001	NA	NA	NA	NA
C	3.12(2.86 to 3.37)	<0.001	NA	NA	NA	NA	2.24(1.95 to 2.52)	<0.001	NA	NA	NA	NA
I vs. C	NA	NA	6.55(6.04 to 7.06)	<0.001	656.366	0.929	NA	NA	8.39(7.92 to 8.87)	<0.001	1263.338	0.962
COPD-Q												
I	2.96(2.14 to 3.79)	<0.001	NA	NA	NA	NA	3.71(2.83 to 4.58)	<0.001	NA	NA	NA	NA
C	1.94(1.24 to 2.65)	<0.001	NA	NA	NA	NA	1.61(0.84 to 2.37)	<0.001	NA	NA	NA	NA
I vs. C	NA	NA	1.33(0.57 to 2.10)	<0.001	12.341	0.198	NA	NA	2.41(1.51 to 3.31)	<0.001	28.837	0.366

I, intervention group; C, control group; PAM-13, Patient Activation Measure; CAT, COPD Assessment Test; Ex-SRES, Exercise Self-Regulatory Efficacy Scale; CSMS, COPD Self-Management Scale; PSSS, Perceived Social Support Scale; COPD-Q, COPD Knowledge Questionnaire; NA, not applicable.

Adherence rates among the PR group significantly declined from 58.82 to 31.37% by 24 weeks post-intervention (*p* < 0.001), while the CC-CoI group maintained stable adherence with no significant change (66.67% vs. 56.86%; *p* = 0.332) (Supplementary Table 3). However, the CC-CoI group demonstrated significantly higher adherence rates at both 12 weeks (*p* = 0.029) and 24 weeks (*p* = 0.010), as illustrated in Figure 3.

### 3.3 Secondary outcome

The CAT scores decreased in both groups by 24 weeks, with the CC-CoI group showing a more pronounced reduction (MD = −3.08; *p* < 0.001) than the PR group (MD = −1.47; *p* = 0.276). Although the reduction was greater in the CC-CoI group, the between-group difference was not significant at 24 weeks (*F* = 3.75, *p* = 0.058, *η*^2^ = 0.07) (Table 3). The CC-CoI group, however, saw a significant increase in the proportion of patients with a CAT score improvement of more than 2 points by 24 weeks (80.39% vs. 54.90%; *p* = 0.002), compared to the PR group (37.25% vs. 50.98%; *p* = 0.143), with a significant between-group difference at this time point (*p* = 0.002).

The mMRC score significantly decreased in the CC-CoI group (*p* < 0.001), with no significant change in the PR group (Supplementary Table 3). The CC-CoI group also showed a significant improvement in Ex-SRES scores at 24 weeks (MD = 12.29, *p* < 0.001), compared to a smaller increase in the PR group (MD = 5.22, *p* < 0.001), with a significant between-group difference (*F* = 84.01, *p* < 0.001, *η*^2^ = 0.63) (Table 2). The Ex-SRES score trends over time are shown in Figure 3, with a significant group-by-time interaction effect (*F* = 66.94, *p* < 0.001, *η*^2^ = 0.57) (Table 3).

The CC-CoI group experienced a significant improvement in CSMS scores at all post-test time points (MD = 36.63, *P* < 0.001), while the PR group’s scores improved initially at 12 weeks (MD = 9.90, *p* < 0.001) but declined by 24 weeks (MD = 8.39, *P* < 0.001) (Table 2). The CSMS score trends over time are presented in Figure 3, with a significant group-by-time interaction effect (*F* = 169.06, *P* < 0.001, *η*^2^ = 0.77) (Table 3).

Perceived social support significantly increased in the CC-CoI group compared to the PR group at both 12 weeks (*F* = 656.37, *p* < 0.001, *η*^2^ = 0.93) and 24 weeks (*F* = 1263.34, *p* < 0.001, *η*^2^ = 0.96). The CC-CoI group consistently showed an increase in perceived social support (MD = 10.06, *p* < 0.001), while the PR group’s scores improved initially but declined by 24 weeks (MD = 2.24, *p* < 0.001) (Table 2). Figure 3 shows the trends in perceived social support, with a significant group-by-time interaction effect (*F* = 397.96, *P* < 0.001, *η*^2^ = 0.89) (Table 3).

Lastly, the CC-CoI group demonstrated a significant increase in COPD-Q scores from baseline to 24 weeks (MD = 3.71, *p* < 0.001), compared to the PR group’s improvement of 1.61 points (p < 0.001). Significant between-group differences were observed at both 12 and 24 weeks (*F* = 12.34, *p* < 0.001, *η*^2^ = 0.20; *F* = 28.84, *p* < 0.001, *η*^2^ = 0.37, respectively) (Table 2), with a significant group-by-time interaction effect (*F* = 11.29, *p* < 0.001, *η*^2^ = 0.18) (Table 3). The COPD-Q score trends are depicted in Figure 3. At the end of the 12-week intervention, the CC-CoI group reported a higher satisfaction score than the PR group (MD = 2.31, *p* = 0.021).

## 4 Discussion

### 4.1 Findings

Our study demonstrated that the CC-CoI platform significantly improved activation, self-efficacy, self-management skills, perceived social support, and adherence to Tele-PR exercise among older adults with COPD. It relieved dyspnea symptoms and improved quality of life, suggesting a safe, feasible, and effective form of Tele-PR intervention. The findings support the role of theory-based co-creation in bolstering intervention effectiveness, highlighting its broad applicability in chronic disease management.

The results of this study demonstrated that the proportion of patients with high adherence in the intervention group was significantly greater than in the control group. This indicates that incorporating the co-creation component into the Tele-PR intervention positively influences the exercise rehabilitation adherence among older adults with COPD. This finding is consistent with the results of the study by Halvorsrud et al. (29). Similarly, research on diabetic patients conducted by Golin et al. (59) supported that enhancing patients’ comprehension of the content of the rehabilitation program may indirectly elevate adherence. Furthermore, Picorelli et al. (60) demonstrated that older adults’ adherence to rehabilitation exercises is affected by their level of disease cognitive. Through co-creating comics, patients have developed a clearer comprehension of health information and the diverse components of rehabilitation programs on the platform. In the ‘Small Tarn West of the Knoll’ module, the CC-CoI platform has boosted patients’ disease knowledge, thereby improving adherence to PR and ensuring treatment efficacy. Meanwhile, self-efficacy is also an important predictor of behavioral change. This study improved patients’ skills and problem-solving techniques through the use of cartoon images, co-creating comics, and other gamified formats. These activities allowed patients to more intuitively recognize their own progress, thereby enhancing their self-efficacy. Norouzkhani et al. (61) demonstrated that integrating self-management tasks for inflammatory bowel disease patients into gamified frameworks, such as point systems and rewards, enhances their sense of accomplishment and self-efficacy. Batch et al. (62) reported that using game-based mechanisms, such as feedback and challenges, to motivate diabetes patients through 12 task levels significantly improved their self-efficacy in self-management. In conclusion, integrating “co-creation” and “gamification” into Tele-PR can significantly improve rehabilitation outcomes and foster positive behavior change, thereby optimizing chronic disease management.

The results of this study indicated that the CC-CoI platform effectively alleviated patients’ breathing difficulties, which were closely linked to ensure the correctness of the patients’ daily rehabilitation training. Studies have demonstrated that exercise training plays a crucial role in the effectiveness of rehabilitation. From a pathophysiological mechanism perspective, standardized pulmonary rehabilitation training can directly address the small airway obstruction that causes breathing difficulties in COPD patients. This is achieved by enhancing the strength of respiratory muscles, such as the diaphragm, and improving the ventilation efficiency of small airways. Previous studies have sought to standardize rehabilitation exercises for COPD patients using wearable devices, particularly chest-worn devices that monitor exercise intensity and frequency during daily activities (63). However, widespread adoption of these devices among older adults with COPD remains limited. Owing to the diverse characteristics of exercise rehabilitation movements, wearable devices struggle to precisely identify the standardization of various movements. Additionally, problems such as poor wearing comfort and the leakage of privacy in exercise videos exist. Nevertheless, the ‘Ageless Atrium’ module effectively overcomes these limitations by employing an interactive feedback mechanism for co-creating comics. To ensure the accuracy of patients’ daily training, this research co-created the comic titled “Exercise to Move” as part of the ‘Ageless Atrium’ module. During the co-creating comics process, medical staff can promptly assess whether patients have mastered and applied pulmonary rehabilitation knowledge and whether their exercise movements are correct, using keywords input by the patients and descriptions of the rehabilitation actions. If movement errors are identified, a group discussion may be convened to correct them promptly. If patients forget the prescribed rehabilitation movements, they can use the video materials linked to the clickable area of the module for a rapid review, thereby improving rehabilitation outcomes further. This design also aligns with the perspective proposed by Park et al. (63) that non-invasive rehabilitation monitoring tools with high acceptability should be developed. However, this study found that the platform had no significant effect on CAT scores. As a complex and comprehensive indicator (64), CAT scores are influenced not only by shortness of breath but also by physiological, psychological, and environmental factors, many of which may not be fully addressed through remote interventions incorporating co-creation features. Secondly, the meta-analysis by Huang et al (31). reported that co-creation has a moderate positive effect on health-related outcomes (e.g., body mass index, blood pressure, and mortality rate), although the results showed substantial heterogeneity. Similarly, the meta-analysis by Halvorsrud et al. (29) found that the effect of co-creation on health outcomes was relatively small (0.25). These findings partially support the conclusions of our study. Furthermore, this study found that, by the 24th week, the proportion of patients in the intervention group whose CAT score improved by more than 2 points was significantly higher, while no significant change was observed in the control group. This suggests that the intervention group experienced a more pronounced improvement in the health status of older adults with COPD compared to the control group. However, the effects of the CC-CoI platform may require a longer follow-up period to be fully realized. Future research should more explicitly examine this temporal relationship and consider incorporating a biofeedback mechanism to address various aspects of patients’ quality of life more directly.

Additionally, the findings of this study confirmed that the training platform significantly improved the activation of older adults with COPD. Increased activation is strongly linked to the co-creation process. Similarly, some other researches also demonstrated that cooperative partnership formed by co-creation foster skills, confidence and experiences among participants, ultimately improving motivation (65, 66). Notably, research on co-creation in chronic disease management suggests that developing intervention strategies align with patients’ interests is accomplished by stakeholders through consultation on patient needs and perspectives. These efforts are largely confined to “say” and “do,” failing to materialize “make” (19, 67, 68), which involves patients expressing their ideas to others through the work. Previous studies have demonstrated that expressing one’s thoughts can increase participants’ engagement. Ravaccia et al. (69) reported that the use of digital support tools, which create a space for users to freely communicate and share their thoughts and experiences, effectively stimulates their willingness to seek help and support others, thereby enhancing their motivation to address psychological issues. Stoica et al. (70) also observed, in their exploration of emotional language during a state of reflective rest, that enabling cognitively intact older adults to express their thoughts freely via the “think-aloud” method effectively increased their propensity to use positive language. At the psychological level, this shift indirectly enhanced the older adults’ initiative to actively exhibit positive cognitive patterns. The co-creation design in this study addresses the absence of the “make” component in traditional models. In the ‘Ageless Atrium’ module, under the organization and guidance of nurses, patients can express their thoughts based on the multiple images generated by the platform. They combine their own disease-living experiences, the effects of exercise rehabilitation, and their medication-related doubts. The design of different thematic scenarios not only intuitively reduces patients’ difficulty in understanding COPD rehabilitation knowledge but also encourages them to share personal experiences and problems, thus stimulating emotional resonance among them. Additionally, this module uses the Socratic method (71) to foster patient creativity and understanding in the form of digital labor, which promotes knowledge sharing and equal collaboration. Digital labor refers to activities on platforms like social media that seem to be purely for leisure and entertainment yet inherently generate value creation (28, 72). The ‘Ageless Atrium’ module offered frequent opportunities for patients to share knowledge and participate in interactive dialog through co-creating an accurate and meaningful comic. This increased motivation for consistent PR exercise completion.

Self-management is essential for individuals with chronic illnesses and significantly impacts health outcomes (22). While telephone coaching has demonstrated efficacy in enhancing self-management among patients with mild COPD (22), the majority of interventions continue to depend on conventional education and skills training, overlooking the potential for co-creation. Heijmans et al. (79) identified that teaching models based solely on one-way knowledge transfer lack interactive feedback, which hinders patients’ ability to effectively apply relevant self-management skills when their condition recurs. Our study significantly enhanced patients’ self-management capabilities through the facilitation of interaction at the instructional, social, and cognitive levels via the co-creating comics in the ‘Ageless Atrium’ module. Srulovici et al. conducted a study to evaluate the effectiveness of the Diabetes Conversation Map™ Program. Their findings indicated that when facilitated by nurses, this program exerted a positive impact on patients’ clinical outcomes and health behaviors (71). Additionally, Qasim et al. (73) reported that educational interventions based on the Diabetes Conversation Map demonstrated significantly greater effectiveness in improving patients’ dietary control and blood glucose monitoring capabilities compared to conventional care. In our study, the ‘Small Tarn West of the Knoll’ module develops self-rescue and self-management skills characteristic of a smart patient by evolving diagnostic data into understandable risk factors and rehabilitation knowledge (74, 75). The ‘Ageless Atrium’ module enhances their deeper understanding of PR-related knowledge and self-management skills, utilizing co-creating comics in an environment that fosters open expression. The ‘Exploitation of Innovation’ module supports patients in applying their acquired knowledge to real-life scenarios through the practical engagement in the virtual scene. This study introduces a novel method for enhancing self-management in chronic disease contexts.

Social support offers robust emotional and resource stability, facilitating behavioral changes among older adults (76). This study indicated that the CC-CoI platform increased patients perceived social support via co-creation, reflecting the emotional and resource support patients received. Firstly, the ‘Ageless Atrium’ module enables patient connectivity on a social platform via co-creating comics, which promotes the exchange of knowledge and emotions, facilitates problem discussion, and aids in resolving operational challenges. The co-creation process bolsters patient social interaction, aids in building virtual communities, and increases the level of perceived social support among patients. Leask et al. (77) suggest that co-creation in research may bolster community relations, aligning with this study’s findings. Secondly, the co-creation within the platform not only enables patients to actively seek social support, but also transforms them from passive recipients to active contributors. Notably in this study, real-time medical guidance and feedback further solidify a cohesive support system, as evidenced by the sustained increase in perceived social support even 3 months post-intervention. Currently, virtual communities have emerged as the main platform for online engagement among older adults (78), and the behaviors exhibited by this demographic in such settings merit observation. The study reveals that older adults in virtual communities instinctively participate in reciprocal and altruistic behaviors. The emergence and evolution of virtual communities constitute a dynamic and gradual process. Initially, individuals start by familiarizing themselves with each other, a process that incrementally builds a network of community relationships. Through co-creation, older adults’ participation in decision-making is amplified (17). They personally experience the benefits brought by the virtual community, which in turn increases patient adhesion to the community. This heightened involvement strengthens the sense of belonging among patients, ultimately spurring reciprocal and altruistic behaviors. Within the community, mutual aid and altruistic behaviors significantly boost participation motivation and reinforce social support networks.

As artificial intelligence (AI) technology continues to evolve and mature, embedded AI is anticipated to be integrated into existing health management platforms. By enhancing the interaction logic and personalizing the adaptation capabilities of digital co-creation functions, AI will offer more precise technical support for the development and maintenance of health behaviors in older adults. In this context, the core objective of remote chronic disease management will further focus on empowering patients. Specifically, intervention strategies should not only be utilized to cultivate “active patients” with proactive health awareness, but also efforts should be dedicated to guiding them to evolve into “smart patients” who can interpret health data and make independent health-related decisions. This developmental pathway not only aligns with the technological evolution trend in the digital health domain, but also provides a crucial guarantee for the long-term management of older populations’ health behaviors in an aging society, while further pointing out an important direction for subsequent research and practical exploration.

### 4.2 Application

This study introduces a novel approach to managing COPD patients, utilizing tele interventions that are based on the co-creation and CoI framework. The study not only enhances behavioral intervention strategies for Tele-PR but also broadens the potential applications of remote medical interventions in chronic disease management. Furthermore, this research offers a pivotal method and tool for digital interventions among older adults communities. The tele rehabilitation platform, by creating virtual communities and incorporating co-creation modules, provides older adults with an easier rehabilitation route and social support network.

### 4.3 Limitations

This study has certain limitations. Firstly, the study’s regional sample from a Southeastern Chinese city may not represent the broader COPD patient populations in other regions or countries. Meanwhile, the patients included in this study have the problem of an unbalanced gender distribution. Future studies should employ larger, randomized samples to validate the platform. Secondly, Tele-PR adherence was assessed via subjective methods, potentially misrepresenting actual exercise engagement. Future studies may integrate digital indicators (e.g., application usage patterns or exercise completion timestamps) to enhance the objectivity of adherence-related data. Thirdly, this study did not conduct a detailed item analysis of the CAT scores. Future research should explore the response patterns of different symptom dimensions to the intervention measures, in order to achieve more profound findings. Fourthly, the 12-week follow-up is inadequate for assessing long-term efficacy. Extended follow-up is needed for comprehensive evaluation of the CC-CoI platform in chronic disease management.

## 5 Conclusion

This study demonstrates that digital co-creation enhances the active participation of older adults in Tele-PR and fosters the development of virtual communities along with the onset of altruistic behaviors in individuals. This approach encourages patients to apply their knowledge for self-management of health behaviors, which in turn, enhances their adherence to rehabilitation exercise and leads to better health outcomes. Furthermore, the platform’s digital co-creation capabilities can be enhanced with embedded AI to promote healthy behaviors in older adults.

## Data Availability

The raw data supporting the conclusions of this article will be made available by the authors, without undue reservation.
