# Supplementary material for: Effectiveness of digital co-creation platform in remote pulmonary rehabilitation for older adults with chronic obstructive pulmonary disease: a randomized controlled trial
Source: Front Public Health. 2025 Nov 10;13:1708607. doi: 10.3389/fpubh.2025.1708607 (PMC12640875; doi:10.3389/fpubh.2025.1708607)
Supplement: Supplementary file 1 [file Table_1.DOCX]

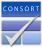
**CONSORT 2010 checklist of information to include when reporting a randomised trial***

| **Section/Topic** | **Item**  **No Checklist item** | **Reported on page No** |
| --- | --- | --- |

**Title and abstract**

1a Identification as a randomised trial in the title Page 1

1b Structured summary of trial design, methods, results, and conclusions (for specific guidance see CONSORT for abstracts) Page 1

**Introduction**

Background and 2a Scientific background and explanation of rationale Page 1-2

objectives 2b Specific objectives or hypotheses

Page 1-2

**Methods**

Trial design 3a Description of trial design (such as parallel, factorial) including allocation ratio Page 3

3b Important changes to methods after trial commencement (such as eligibility criteria), with reasons Page 4

Participants 4a Eligibility criteria for participants Page 4

4b Settings and locations where the data were collected Page 4

Interventions 5 The interventions for each group with sufficient details to allow replication, including how and when they were

actually administered Page 3-7

Outcomes 6a Completely defined pre-specified primary and secondary outcome measures, including how and when they

were assessed Page 7-8

6b Any changes to trial outcomes after the trial commenced, with reasons

Sample size 7a How sample size was determined Page 4

7b When applicable, explanation of any interim analyses and stopping guidelines

Randomisation:

Sequence 8a Method used to generate the random allocation sequence Page 3

generation 8b Type of randomisation; details of any restriction (such as blocking and block size) Page 3

Allocation 9 Mechanism used to implement the random allocation sequence (such as sequentially numbered containers),

concealment describing any steps taken to conceal the sequence until interventions were assigned

mechanism Page 3

Implementation 10 Who generated the random allocation sequence, who enrolled participants, and who assigned participants to

interventions Page 3

Blinding 11a If done, who was blinded after assignment to interventions (for example, participants, care providers, those


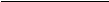
assessing outcomes) and how Page 3

11b If relevant, description of the similarity of interventions

Statistical methods 12a Statistical methods used to compare groups for primary and secondary outcomes Page 8

12b Methods for additional analyses, such as subgroup analyses and adjusted analyses Page 8

**Results**

Participant flow (a 13a For each group, the numbers of participants who were randomly assigned, received intended treatment, and

diagram is strongly were analysed for the primary outcome Page 8-9

recommended) 13b For each group, losses and exclusions after randomisation, together with reasons Page 8-9

Recruitment 14a Dates defining the periods of recruitment and follow-up Page 3,8-9

14b Why the trial ended or was stopped Page 8-9

Baseline data 15 A table showing baseline demographic and clinical characteristics for each group Page 22

Numbers analysed 16 For each group, number of participants (denominator) included in each analysis and whether the analysis was

by original assigned groups Page 9-10

Outcomes and 17a For each primary and secondary outcome, results for each group, and the estimated effect size and its

estimation precision (such as 95% confidence interval) Page 9-10

17b For binary outcomes, presentation of both absolute and relative effect sizes is recommended Page 9-10

Ancillary analyses 18 Results of any other analyses performed, including subgroup analyses and adjusted analyses, distinguishing

pre-specified from exploratory Page 9-10

Harms 19 All important harms or unintended effects in each group (for specific guidance see CONSORT for harms) Page 8-9

**Discussion**

Limitations 20 Trial limitations, addressing sources of potential bias, imprecision, and, if relevant, multiplicity of analyses Page 13

Generalisability 21 Generalisability (external validity, applicability) of the trial findings Page 10-13

Interpretation 22 Interpretation consistent with results, balancing benefits and harms, and considering other relevant evidence Page 10-14

**Other information**

Registration 23 Registration number and name of trial registry Title page

Protocol 24 Where the full trial protocol can be accessed, if available

Funding 25 Sources of funding and other support (such as supply of drugs), role of funders · Title page

*We strongly recommend reading this statement in conjunction with the CONSORT 2010 Explanation and Elaboration for important clarifications on all the items. If relevant, we also

recommend reading CONSORT extensions for cluster randomised trials, non-inferiority and equivalence trials, non-pharmacological treatments, herbal interventions, and pragmatic trials. Additional extensions are forthcoming: for those and for up to date references relevant to this checklist, see www.consort-statement.org.
