# Supplementary material for: Effectiveness of digital co-creation platform in remote pulmonary rehabilitation for older adults with chronic obstructive pulmonary disease: a randomized controlled trial
Source: Front Public Health. 2025 Nov 10;13:1708607. doi: 10.3389/fpubh.2025.1708607 (PMC12640875; doi:10.3389/fpubh.2025.1708607)
Supplement: Supplementary file 2 [file Table_2.DOCX]

**Table of Contents**

[**1.Executive Summary 2**](#_Toc184749139)

[**2.Development of CoI-CC Platform 2**](#_Toc184749140)

[**3.Model Design 3**](#_Toc184749141)

[**4.Method 3**](#_Toc184749142)

[**5.Participants 4**](#_Toc184749143)

[**6.Core task 4**](#_Toc184749144)

[**7.Test Tools 4**](#_Toc184749145)

[**8.Result 5**](#_Toc184749146)

# 1.Executive Summary

The objective of this test was to document the development process (Alpha and Beta testing) and the gained insights, to make this knowledge available to other developers and scientists. The user experience was investigated through user interview. This test provided evidence of the usability of digital co-creation (CC-CoI) platform.

# 2.Development of CoI-CC Platform

Within the present study the first four phases (0–3) of the mHealth Agile Development & Evaluation Lifecycle were completed. Figure ​1 illustrates the lifecycle, adapted to the CoI-CC platform development project.


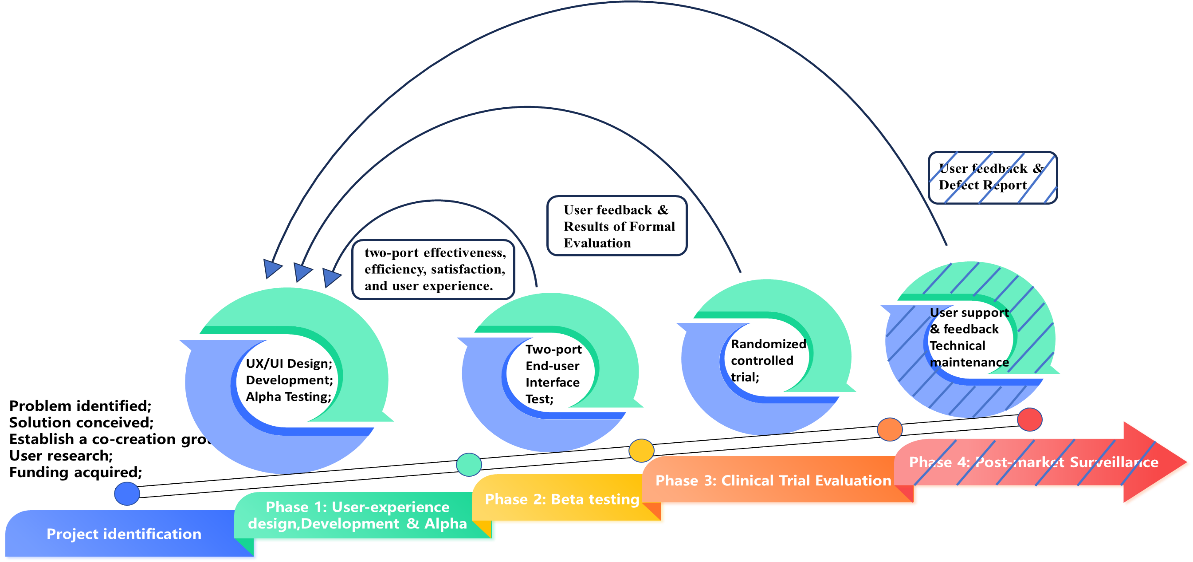


Figure ​1 The mHealth Agile Development & Evaluation Lifecycle of CoI-CC platform

In phase zero, the project identification phase, we included various stakeholders (respiratory specialists, clinicians, rehabilitation therapists, nurses, software engineers, user interface designers, and older adults with COPD) and conducted interviews to understand the obstacles of participants’ low adherence with Tele-PR programs and key expectations for the platform. In phase one, the development and Alpha testing of the platform, we focused on the platform’s acceptance and usability among stakeholders and the preparation of a two-port patient interface for Beta testing. In phase two, the Beta testing, the focus is on in-depth testing, providing two-port effectiveness, efficiency, satisfaction, and user experience. In phase three, the platform’s effectiveness was validated through a randomized controlled trial.

# 3.Model Design

**1) ‘Ageless Atrium’**

Table 1. Thematic comic scene-keyword mapping chart

| Scene | Keyword |
| --- | --- |
| Living with COPD | Breathing exercises (boxing, exercise); An inhaler in your pocket; Portable oxygen machine; Community health station; Community education resources (health talks); Vaccination; Dress warmly; A face mask; No smoking; Air quality monitoring; Public transport (vehicle exhaust); Family cares (neighbors)... |
| Exercise to Move | Uppers; Chest Expansion; Wall Push; Kickers; Shakers; Stretch Band Flat Pulls; Stretch Band Upper Pulls; Stretch Band Incline Pulls; Internal Rotation; Lateral Upper Extremities; Upper Extremities Flat Lifts; Upper Extremities Upper Lifts; Leg Raises; Heel-Toe Walks; Tiptoe Exercises... |
| Safety Lessons from the Medicine Cabinet | Budesonide/Formoterol Symbicort; Salmeterol Xinafoate and Fluticasone Propionate Aerosol; Tiotropium bromide; Trelegy Ellipta; Theophylline; Antitussive; Expectorant Chinese medicine preparation… |
| COPD and Caring for Your Heart | Morning heart rate monitoring; A nutritious breakfast; Exercise (sports); Quit smoking; Take medicine on time; Doctor's consultation; Family support; Stress management; Regular examination (electrocardiogram); Community Education Resources (Health Talk) ... |

**2) ‘Exploitation of Innovation’**

Table 2. Core scenes design and risk factors

| Scene | Risk factor | Set task |
| --- | --- | --- |
| Eco-serenity Oasis | Cold winds, air pollution, dust | 1. The character can start from the lower left corner of the scene to move to the community health site, avoiding risk factors in the middle. |
| Vogue Plaza Delight | Chili, fried food, sashimi | 1. The character can move the forbidden food in the shopping cart to the shelf. |
| Culinary Innovation Hub | Oil smoke, BMI > 30 characters, smoking | 1. The character can move from the top right corner of the scene to the dining table, avoiding the danger factor halfway.  2. Click on the person with BMI > 30 and choose the appropriate words to tell him. |
| Living Sphere | Smoking, flowers, colds and sneezes | 1. The character can move from the bottom left corner of the scene to the sofa, avoiding the danger factor. |

# 4.Method

This study used a mixed method to test the CC-CoI platform. After participant recruitment, the research assistant instructed them to complete a predefined core task while observing their task completion. Participants then completed questionnaires and participated in user interviews.

# 5.Participants

Table 3. Characteristics of the participants (n=20)

| Measures | Mean (SD) / n% |
| --- | --- |
| **Sex** |  |
| Male | 16(80) |
| Female | 4(20) |
| **Age,** **years** | 71.94±5.86 |
| **Education** |  |
| Primary school | 4(20) |
| Middle school | 9(45) |
| High school | 3(15) |
| Technical school | 3(15) |
| College or postgraduate | 1(5) |
| **Monthly income, RMB, ¥** |  |
| ＜5000 | 14(70) |
| ≥5000 | 6(30) |
| **Social status (living alone)** |  |
| Yes | 4(20) |
| No | 16(80) |

# 6.Core task

Table 4. Core tasks design

| Number | Name | Description |
| --- | --- | --- |
| Task 1 | Achieve your PR learning goals | Mark your daily schedule in the ‘Day trip’ module and click on the visual learning map to learn PR knowledge in the ‘Small Tarn West of the Knoll’ module. |
| Task 2 | Co-creating comics | Co-create comics using the text-to-image tool. |
| Task 3 | Dispose of risk factor cartoons | Move the cartoon images of the risk factor into the trash bin. |
| Task 4 | Rehabilitation report | Report the number and duration of rehabilitation exercise this week. |

# 7.Test Tools

The following tools were used in Alpha testing:

1. The Acceptability questionnaire

2. The System Usability Scale (SUS)

The following tools were used in Beta testing

1. Task completion rate

2. Customer Efforts Score (CES)

3. User Satisfaction Questionnaire (USQ)

4. Net Promoter Score (NPS)

5. Outline of user experience interview

# 8.Result

**Results of Alpha testing**

1) The Acceptability questionnaire

Table 5. Results of the acceptability questionnaire

| Questionnaire item | Yes (n) | | | |
| --- | --- | --- | --- | --- |
| This CoI-CC platform is simple to operate and clear to navigate. | Older adults with COPD | HCPs | Caregiver | Total/composition ratio |
| The content is clear and easy to understand. | 9 | 4 | 5 | 18/100% |
| Material of suitable length | 8 | 3 | 4 | 15/83% |
| Sentence length | 7 | 4 | 4 | 15/83% |
| Videos and explanations in the platform are very helpful. | 8 | 3 | 5 | 16/88% |
| Is the time taken to complete this CoI-CC platform acceptable? | 6 | 3 | 2 | 11/61% |
| The font size and background color of the platform are appropriate. | 7 | 4 | 4 | 15/83% |
| The platform provides support for medical staff and families. | 8 | 4 | 3 | 15/83% |
| The platform helps improve COPD knowledge and self-management skills. | 7 | 3 | 3 | 13/72% |
| Please rate your level of usefulness of the ‘Small Tarn West of the Knoll’ module. | 8 | 4 | 5 | 17/94% |
| Please rate your level of usefulness of the ‘Ageless Atrium’ module. | 9 | 3 | 4 | 16/88% |
| Please rate your level of usefulness of the ‘Exploitation of Innovation’ module. | 7 | 3 | 5 | 15/83% |
| Overall, how satisfied are you with the platform? | 7 | 3 | 4 | 14/78% |

2) SUS


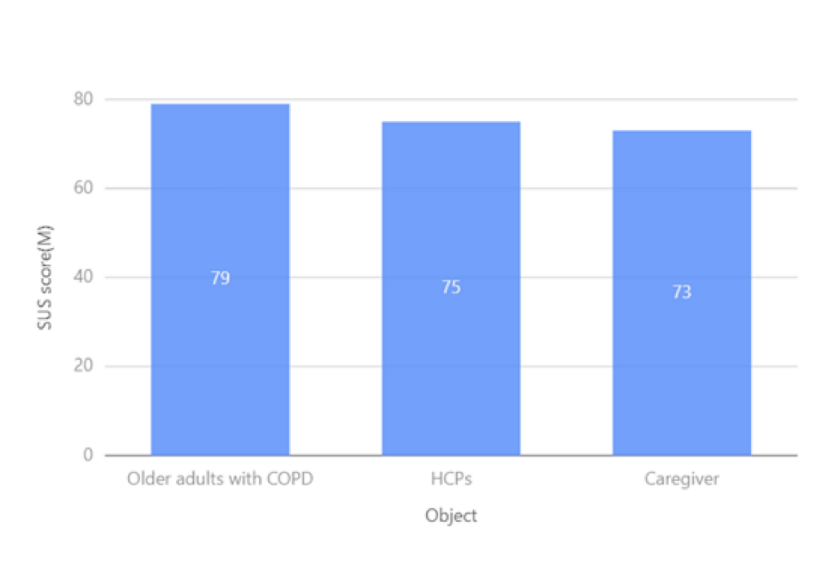


Figure ​2. Results of SUS

**Results of Beta testing**

1) Task completion rate, CES, USQ, NPS

Table 6. Results of Beta testing

| Issues |  | n% /、 n |
| --- | --- | --- |
| Task completion rate |  | 100 |
| CES |  | 3.30 |
| USQ |  |  |
|  | Usability | 4.0 |
|  | Interfacial design | 4.2 |
|  | Content quality | 4.4 |
|  | Technology acceptance | 3.8 |
|  | Overall experience | 4.3 |
| NPS |  | 4.60 |

2) User Interview

Table 7. Results of user interview

| User experience problem | User insight |
| --- | --- |
| Satisfaction with the treatment content | - Have a positive attitude towards text and video content. - After use, we can understand the prevention of COPD recurrence and improve the compliance of exercise rehabilitation exercise. - I cannot see my previous training. It is recommended to add the record function. |
| Satisfaction with the form of treatment | - Some participants expressed appreciation for the free format, that there were no difficulties during the discussion and that technology was acceptable. - One participant was stressed by not knowing how much energy to expend. |
| User friendliness | - Some participants found the layout and examples helpful. - The design, format, and information content of the training platform are user-friendly to participants, but lack a clear path to guide participants through all treatments. |
| The degree of stimulation of treatment | - Many participants found the training very motivating. - Some participants emphasized that it was more motivating to receive support from other patients. - It is recommended to emphasize that treatment progress requires repeated participation of participants, such as repeated exercise. |
