# Supplementary material for: Effectiveness of digital co-creation platform in remote pulmonary rehabilitation for older adults with chronic obstructive pulmonary disease: a randomized controlled trial
Source: Front Public Health. 2025 Nov 10;13:1708607. doi: 10.3389/fpubh.2025.1708607 (PMC12640875; doi:10.3389/fpubh.2025.1708607)
Supplement: Supplementary file 3 [file Table_3.DOCX]

Supplemental material 4

Within-group differences and between-group differences of outcomes (mMRC, Adherence rate).

| Measures | Intervention | | Control | | Difference between groups | |
| --- | --- | --- | --- | --- | --- | --- |
|  | Median (range) / n% | P | Median (range) / n% | P | $\chi^{2}$/ Z | P |
| **mMRC** |  |  |  |  |  |  |
| T1 | 2(1,2) | ＜0.001 ^d^ | 2(2,3) | NA | -4.431 | ＜0.001 ^a^ |
| T2 | 2(1,3) | ＜0.001 ^d^ | 2(2,3) | 0.058 ^e^ | -2.852 | 0.004 ^a^ |
| **Adherence rate ≥ 75%** |  |  |  |  |  |  |
| T1w | 34(67) | NA | 30(59) | NA | 0.671 | 0.413 ^c^ |
| T1 | 31(61) | 0.607 ^b^ | 20(39) | 0.006 ^b^ | 4.745 | 0.029 ^c^ |
| T2 | 29(57) | 0.332 ^b^ | 16(31) | ＜0.001 ^b^ | 6.720 | 0.010 ^c^ |

Abbreviations: T1w, 1-week; T1, 12-week; T2, 24-week; mMRC, modified Medical Research Council scale; NA, not applicable.

^a^Mann-Whitney U test; ^b^McNemar test; ^c^Chi-squared test; ^d^Wilcoxon rank-sum test; ^e^Friedman’s ranktest.
